# Ly9 (CD229) Cell-Surface Receptor is Crucial for the Development of Spontaneous Autoantibody Production to Nuclear Antigens

**DOI:** 10.3389/fimmu.2013.00225

**Published:** 2013-07-31

**Authors:** Jose de Salort, Marta Cuenca, Cox Terhorst, Pablo Engel, Xavier Romero

**Affiliations:** ^1^Immunology Unit, Department of Cell Biology, Immunology and Neurosciences, Medical School, University of Barcelona, Barcelona, Spain; ^2^Division of Immunology, Beth Israel Deaconess Medical Center, Harvard Medical School, Boston, MA, USA; ^3^Institut D’Investigacions Biomèdiques August Pi i Sunyer (IDIBAPS), Barcelona, Spain

**Keywords:** SLAMF, Ly9 (CD229, SLAMF3), anti-DNA autoantibodies, disease susceptibility, systemic lupus erythematosus, murine Lupus

## Abstract

The *Signaling Lymphocyte Activation Molecule Family* (*SLAMF*) genes, which encode cell-surface receptors that modulate innate and adaptive immune responses, lay within a genomic region of human and mouse chromosome 1 that confers a predisposition for the development of systemic lupus erythematosus (SLE). Herein, we demonstrate that the SLAMF member Ly9 arises as a novel receptor contributing to the reinforcement of tolerance. Specifically, *Ly9*-deficient mice spontaneously developed features of systemic autoimmunity such as the production of anti-nuclear antibodies (ANA), -dsDNA, and -nucleosome autoantibodies, independently of genetic background *[(B6.129) or (BALB/c.129)]*. In aged (10- to 12-month-old) *Ly9*^−/−^ mice key cell subsets implicated in autoimmunity were expanded, e.g., T follicular helper (Tfh) as well as germinal center (GC) B cells. More importantly, *in vitro* functional experiments showed that Ly9 acts as an inhibitory receptor of IFN-γ producing CD4^+^ T cells. Taken together, our findings reveal that the Ly9 receptor triggers cell intrinsic safeguarding mechanisms to prevent a breach of tolerance, emerging as a new non-redundant inhibitory cell-surface receptor capable of disabling autoantibody responses.

## Introduction

Systemic lupus erythematosus (SLE) is a multisystem autoimmune disease characterized by major immunological stigmata, the production of antibodies against own cellular nuclear components. The mechanisms underlying SLE are complex and include genetic, epigenetic, environmental, hormonal, and immunoregulatory factors. Furthermore, multiple pathogenic pathways likely contribute to end-organ damage in this heterogeneous disease (1–3). Elucidation of these pathways, as well as the identification of new molecular disease markers and targets that account for the pathogenesis of lupus, are crucial to improve currently available therapeutic approaches.

Genetic predisposition is a central factor to the development of SLE (4, 5). Both Genome Wide Association studies (GWAS) and the in depth analyses of genetically altered and congenic mice have provided insights into the genetic control of lupus. For instance, mouse strains bearing either a NZW- or 129-derived genomic segment that is embedded in chromosome 1 of B6 mice, develop a lupus-like disease due to the epistatic interactions between the 129-derived genes and B6 genes (6). Analysis of congenic NZW × B6 mice identified the Sle1 region of chromosome 1 and its subregions Sle1a, -b, and -c, as key elements involved in breaching B- and T-cell tolerance to chromatin, an essential early step leading to full-blown onset of the disease. Remarkably, multiple gene-wide linkage scans in SLE families have also identified a syngenic lupus susceptibility locus (region 1q23) in human chromosome 1 (7, 8).

Precise mapping of the Sle1 region identified a gene interval of 0.9 Mb, termed Sle1b, as the most potent segment involved in the generation of autoantibodies. Interestingly, this segment includes seven signaling lymphocyte activation molecule family (SLAMF) cell-surface receptor genes (9). The proteins encoded by these genes are the best suited candidates for controlling signaling pathways in Sle1 tolerance due to their extensive polymorphic nature and isoform diversity, coupled with their ability to modulate innate and adaptive immunity (10, 11). The SLAMF receptors (SLAMF1–9) are involved in the functional regulation of several immune cell types, including helper and cytotoxic T lymphocytes, NK cells, and macrophages (12–15). These receptors mediate adhesion and regulate cognate T-cell–B-cell interactions, which elicit signal transduction by recruiting SLAM-associated protein (SAP). Importantly, SAP deficiency selectively impairs the ability of CD4^+^ T cells to stably interact with cognate B cells, avoiding differentiation toward T follicular helper cells (Tfh), and the generation of T-dependent B-cell responses (16–18). Consistent with this role, the absence of SAP mitigates autoimmunity in various mouse models of lupus by disrupting the generation of germinal centers (GC) (19, 20). Increasing evidence suggests that a defective response to self-antigens in the periphery can constitute a relevant pathogenic event. More specifically, autoantibodies detected in lupus patients and lupus-prone mice had suffered isotype switching and somatic hypermutation, which facilitated the binding to self-antigens with high affinity, all indicating the involvement of GC pathways in this disease (21, 22). This, therefore, provides a rational for determining not only the exact role played by SLAMF receptors as key drivers of Tfh and GC formation, but also their potential as appealing therapeutic targets for autoantibody-mediated diseases.

While recent reports have identified Slamf6 (Ly108) receptor and its isoforms and Slamf2 (2B4) as contributing to the role played by Sle1b in tolerance (23–25), the involvement of other SLAMF members cannot be excluded. Of particular interest is the Ly9 (CD229, Slamf3) molecule, since a comparative analysis between B6 and the autoimmune congenic strain B6.Sle1b revealed significant differences in its isoforms usage, as well as in the extent of polymorphisms and expression levels (9), with the evidence indicating the possible participation of Ly9 in B6.Sle1b autoimmunity. Briefly, Ly9 expression is restricted to hematopoietic cells, including B and T lymphocytes (26). As has been shown in other SLAMF members, Ly9 functions as a homophilic adhesion receptor and its cytoplasmic tail contains two copies of the conserved tyrosine-based switch motif (ITSM), which is a docking site for the adapter molecules SAP and EAT-2 (27, 28). *Ly9*-deficient mice with a mixed 129 × B6 background exhibited no major T-cell developmental abnormalities and only very mild defects in T-cell responses (29). Recent findings demonstrate the role of Ly9 as a unique inhibitory cell-surface receptor regulating the size of the thymic innate CD8^+^ T-cell pool and the development of *invariant* Natural Killer T (*i*NKT) cells (30). Nonetheless, the functional role of Ly9 in lupus pathogenesis remains unknown. Here, we use *Ly9-*deficient mice, which were generated with 129-derived ES cells and then backcrossed onto B6 or BALB/c backgrounds, in order to determine the role of the Ly9 receptor in autoantibody development.

## Materials and Methods

### Mice

*Ly9^−/−^* mice (129 × B6), generously provided by Dr. McKean (29), were backcrossed onto BALB/c background for 12 generations to generate the *Ly9^−/−^(BALB/c.129)* strain and onto C57BL/6 (B6) background for 12 generations to generate the *Ly9^−/−^(B6.129)* strain. Eight-week-old BALB/c and B6 wild-type mice were purchased from Charles River Laboratories (Saint-Aubin-lès-Elbeuf, France). All mice strains were maintained under specific pathogen-free (SPF) conditions for up to 12 months. Serum samples were collected from the tail vein at 3-, 6-, 9-, and 12-months of age. At 12 months, mice were euthanized, a peritoneal lavage was carried out and kidneys, bone marrow, spleen, thymus, and sera were harvested. Experiments were conducted in compliance with institutional guidelines as well as with national laws and policies.

### Anti-nuclear antibodies analysis

Anti-nuclear antibodies (ANA) titers were determined by indirect immunofluorescence using permeabilized Hep-2 cells. Serum samples were progressively diluted and incubated for 1 h at room temperature on Hep-2 cells followed by Texas Red-conjugated anti-mouse IgG (Jackson Laboratory, Bar Harbor). After washing, the nucleus was stained with 4′,6-diamidino-2-phenylindole (DAPI). Analysis was performed by fluorescence detection using a Nikon Eclipse fluorescent microscope (Nikon, Tokyo).

### Anti-double-stranded DNA and anti-chromatin detection

ELISA assays were performed to quantify levels of anti-double-stranded DNA (anti-dsDNA) and anti-chromatin antibodies in sera of mice. For anti-dsDNA detection, an ELISA was carried out using heat-denatured calf thymus DNA (Sigma Chemical Co., St Louis, MO, USA). dsDNA was coated onto 96-well plates (Corning Costar, Corning, NY, USA) at 10 μg/ml. Purified antibody anti-dsDNA (Clone HpS22, Immunotools, Friesoythe, Germany), used as standard, was serially diluted. Standards and test serums (dilution 1:100) were incubated on plates for 1 h at room temperature. After extensive washing, autoantibodies were detected using a HRP-conjugated anti-mouse IgG (Sigma-Aldrich) and developed with OPD substrate (Sigma-Aldrich).

Anti-chromatin autoantibodies were detected using nucleosome antigen (Arotec Diagnostics Limited, Wellington, New Zealand). The nucleosome antigen was coated on 96-well plates at 3 μg/ml. Serums were diluted 1:100 and incubated for 1 h at room temperature. Autoantibodies against nucleosome were detected using a HRP-conjugated anti-mouse IgG and developed with substrate. All samples were handled simultaneously under the same experimental conditions and results are expressed as OD values.

### IgG isotype detection

Basal serum IgG isotypes were determined by ELISA using purified goat anti-mouse IgG (Sigma-Aldrich) coated 96-well plates. 1:100 diluted mouse serums were incubated for 1 h at room temperature. After extensive washing, IgG isotypes were detected using biotin-conjugated anti-mouse IgG_1_, IgG_2a_, IgG_2b_, and IgG_3_ (Jackson Laboratory). All samples were handled simultaneously under the same experimental conditions and results are expressed as OD values.

### Flow cytometry

Single-cell suspensions were incubated with 20% heat-inactivated rabbit serum before being stained on ice with fluorophore-labeled antibodies against surface molecules using standard methods. Data was acquired using a FACSCanto II (BD Pharmingen, San Jose, CA, USA) flow cytometer and analyzed with either FACSDiva™ (BD Pharmingen) or FlowJo software (Tree Star, San Carlos, CA, USA). The following anti-mouse mAbs were obtained from BD Pharmingen: CD4-FITC, CD11b-PE, CD21-FITC, CD23-FITC, CD24-FITC, CD43-FITC, CD44-FITC, CD62L-FITC, CD69-FITC, CD154-PE, c-Kit-PE, Ter-119-PE, IgM-biotinylated, and CXCR5-biotinylated. The mAbs CD8-FITC, CD11b-FITC, CD25-PE, CD25-FITC, IgM-FITC, B220-FITC, as well as the isotype-matched control Abs, were acquired from ImmunoTools (Friesoythe, Germany). The following mAbs were obtained from BioLegend (San Diego, CA, USA): CD3-FITC, CD4-Pacific Blue, CD8-PE-Cy5, PD1-PE, PD1-PE-Cy7, B220-Pacific Blue, CD41-FITC, and IgD-APC-Cy7. The mAbs CD3-APC, CD5 PE-Cy7, CD229-APC, Sca-1-APC, and GL-7-FITC were purchased from eBioscience (San Diego, CA, USA). Anti-mouse CD138-APC was obtained from R&D Biosystems (R&D System, Wiesbaden, Germany). R-PE labeled murine CD1d tetramer pre-loaded with PBS57 (NIH Tetramer Core Facility, Atlanta, GA, USA) was used to detect *i*NKT cells according to the manufacturer’s instructions. Streptavidin PERCP-Cy5.5 was obtained from BD Pharmingen and streptavidin PE-Cy5 from eBioscience. For intracellular staining with IFN-γ and IL-17, cells were made permeable with an intracellular staining buffer (eBioscience). Anti-IFN-γ-PE (Clone XMG1.2, BD Pharmingen) or anti-IFN-γFITC (XMG1.2; eBioscience), and anti-IL-17-APC (Clone TC11-18H10.1, BioLegend) were used for intracytoplasmic staining.

### Kidney histology and urine assays

Kidneys were fixed with 4% formalin (PBS), dehydrated, and embedded in paraffin. All sections were counterstained with Gill’s hematoxylin (Panreac, Spain), dehydrated with graded alcohol and xylene, and mounted with DPX (VWR International, Radnor, PA, USA). To evaluate IgG-immunocomplex deposits on kidneys, snap-frozen spleens in OCT media (Sakura Finetek Europe B.V., The Netherlands) were cryosectioned (5 μm), blocked with 6% FCS in PBS, and stained with alexa 488-conjugated anti-mouse IgG (Life Technologies Corporation, Invitrogen, Paisley, UK). Images were analyzed using a Nikon Optiphot-2 microscope (Nikon) and acquired with a high-definition color camera (Nikon).

Freshly voided urine samples were tested for proteinuria using Albustix (Siemens Healthcare Diagnostics Inc. Tarrytown, NY, USA).

### *In vitro* cell activation

Splenic lymphocytes were activated with plate-bound anti-CD3 (2 μg/ml) (145-2C11; BD Pharmingen) combined with purified soluble anti-CD28 (1 μg/ml) (37.51; BD Pharmingen). Splenocytes (100,000 cells/well) were cultured in RPMI 1640 medium supplemented with 10% FBS, 100 IU/ml of penicillin, 100 μg/ml of streptomycin, and 2.5 μM of β-mercaptoethanol in a 96-well plate and activated. Supernatants were collected after 72 h of incubation and IFN-γ levels were measured by ELISA. Additionally, after 24 h of activation, cells were collected and the percentages of activation markers (CD25, CD40L) were analyzed by flow cytometry. Th17 cell polarization was carried out according to BioLegend’s protocol. Briefly, single splenocyte suspensions were activated with 2 μg/ml of plate-bound anti-CD3 (clone 145-11; BioLegend), anti-CD28 (5 μg/ml; clone 37.51, BD Pharmingen), TGF-β1 (1 ng/ml, BioLegend), anti-IFN-γ (10 μg/ml; clone XMG1.2, BD Pharmingen), anti-IL-4 (10 μg/ml; clone 11B11; BioLegend), IL-6 (50 ng/ml, Immunotools), and IL-23 (5 ng/ml, BioLegend) over 3 days. Cells were then stimulated for 4–5 h with phorbol myristate acetate (PMA; 50 ng/ml; Sigma-Aldrich) and ionomycin (750 ng/ml; Sigma-Aldrich) in the presence of GolgiStop at 1:1500 (Pharmingen).

### Measurement of cytokines

The amounts of IFN-γ in sera or in cell-culture supernatants were evaluated with commercially available ELISA kits (R&D Systems). Intracellular analysis of cytokines produced by CD4^+^ and CD8^+^ T cells, and *i*NKT cells was carried out by FACS analysis.

Spleen CD4^+^ T cells were isolated by using MACS CD4^+^ T-cell Isolation Kit II (Miltenyi Biotec), following manufacturer’s protocol. Cells were stimulated for 4–5 h with PMA (50 ng/ml), ionomycin (750 ng/ml), and GolgiStop at 1:1500. APC-conjugated anti-CD3 (eBioscience), pacific blue-conjugated anti-CD4 (BioLegend), PE-Cy7-conjugated anti-CD8 (BioLegend), pacific blue-conjugated anti-B220 (BioLegend), and PE-conjugated tetramer-PBS57 (NIH) were used for flow cytometry staining. Cells were then fixed and permeabilized with Foxp3 Staining Buffer (eBioscience) before intracellular staining with alexa fluor 647-conjugated anti-IL-17 (clone TC11-18H10.1; BioLegend), phycoerythrin-conjugated anti-IFN-γ (XMG1.2; BD Pharmingen) or FITC-conjugated anti-IFN-γ (XMG1.2; eBioscience), IL-4 PE-Cy7 (11B11; BD Pharmingen).

### *In vitro* proliferation and viability assays

Splenocytes were resuspended in PBS with 5% FCS (10^6^ cells/mL), and stained with CFSE (Invitrogen) (1 μL CFSE for each 10^6^ cells), during 5 min at room temperature. Then cells were washed with cold PBS with 5% FCS and with cold PBS. After the second wash, lymphocytes were resuspended in RPMI media (10% FCS, and 2.5 μM β-mercaptoethanol). Cells were stimulated with 10 μg/mL F(ab′)_2_ anti-mouse IgM (Jackson ImmunoResearch), or complete media as a negative control.

In order to measure viability, lymphocytes were stained with fluorochrome-labeled antibodies against surface antigens using standard methods. Cells were washed twice in azide-free and serum/protein-free PBS, and stained with Live/Dead Fixable far Red (Invitrogen) following manufacturer’s protocol. Then lymphocytes were stained with eFluor 450-conjugated Annexin V eBioscience during 15 min at room temperature, and washed with RPMI media. Proliferation and viability were assessed 72 h later.

### Statistics

Mann–Whitney tests were used to calculate *p* values for all numeric data.

## Results

### Ly9-deficient mice develop spontaneous autoimmunity

**Figure 1:**
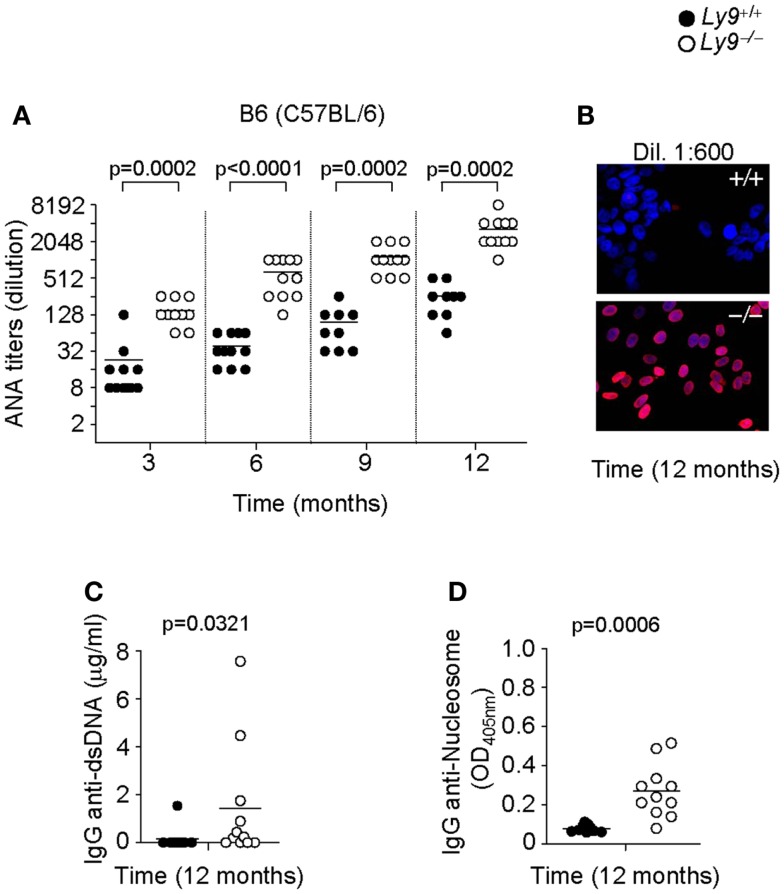
**Autoantibody development in *Ly9^−/−^(B6.129)* mice**. **(A)** Determination of anti-nuclear autoantibody (ANA) titers in the serum from *Ly9*^+/+^ (wt) and *Ly9^−/−^* mice obtained at the indicated time points, and as described in Section “Materials and Methods.” **(B)** Representative immunofluorescence image of permeabilized Hep-2 incubated with sera from 1-year-old wt as compared with 1-year-old *Ly9^−/−^* mice (sera dilution 1:600). After washing, IgG was detected with anti-mouse IgG-Texas Red (red). Nucleus was stained with DAPI (blue). **(C)** ELISA was performed to assess autoantibodies against double-stranded DNA (dsDNA) and **(D)** nucleosome in serum from 12-month-old wt and *Ly9^−/−^* mice (see Materials and Methods). Experiments were initially conducted with a total of *n* = 11 B6 (wt) and *n* = 11 *Ly9^−/−^(B6.129)* female mice. Small horizontal bars indicate the mean. Statistical significances are shown.

To assess the role of Ly9 in humoral autoimmune responses, *Ly9-*deficient mice were generated by mutated 129-derived ES cells (29) and backcrossed with B6 (C57BL/6) or BALB/c mice for 12 generations. First, we analyzed the presence of ANA in sera of 3, 6, 9, and 12 months old *Ly9^−/−^(B6.129)* mice by immunofluorescence staining of Hep-2 cells. As shown in Figures 1A,B, an age-associated increase in ANA titers was detected in *Ly9^−/−^ (B6.129)* mice that was significantly higher than those of their age-matched *Ly9*^+/+^ (wild-type; wt) counterparts. The differences in ANA serum levels between wt and *Ly9^−/−^* mice were statistically significant as early as 3 months of age. At 12 months of age, 80% of the serum samples from *Ly9^−/−^(B6.129)* showed ANA titers ≥1:4096. In contrast, 100% of the serum titers from their wt counterparts measured ≤1:512. Further analysis demonstrated that by age 12 months *Ly9^−/−^(B6.129)* mice displayed significant increases in their circulating levels of anti-double-stranded (ds) DNA and anti-nucleosome IgG antibodies in comparison to their wt counterparts (Figures 1C,D).

**Figure 2:**
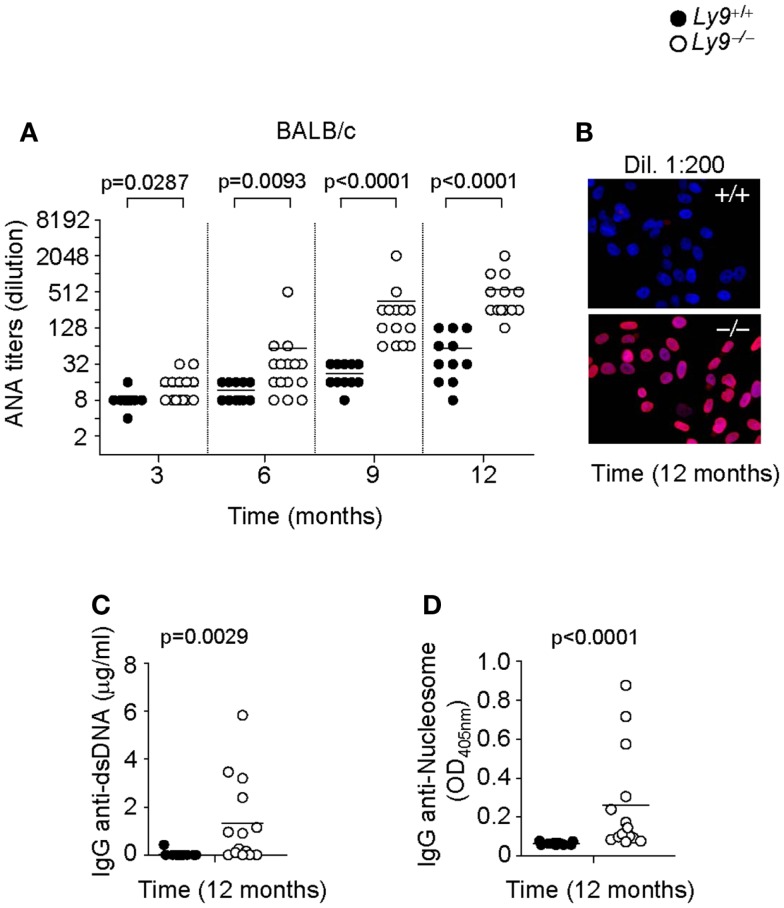
**Spontaneous humoral autoimmune response in *Ly9^−/−^(BALB/c.129)* mice**. **(A)** ANA titers in the serum of 3- to 12-month-old *Ly9*^+/+^ (wt) and *Ly9^−/−^* mice. **(B)** Representative immunofluorescence staining of permeabilized Hep-2 incubated with sera from 1-year-old wt as compared with 1-year-old *Ly9^−/−^* mice (sera dilution 1:200). After washing, IgG was detected with anti-mouse IgG-Texas Red (red). Nucleus was stained with DAPI (blue). **(C)** Determination by ELISA of autoantibodies against double-stranded DNA (dsDNA) and **(D)** nucleosome in serum from 12-month-old wt and *Ly9^−/−^* mice. Experiments were initially conducted with a total of *n* = 11 BALB/c (wt) and *n* = 15 *Ly9^−/−^(BALB/c.129)* female mice. Small horizontal bars indicate the mean. Statistical significances are shown.

In order to further dissect the role of Ly9 in the humoral autoimmune response, excluding any effects stemming from epistatic interactions, we determined autoantibodies in the serum of *Ly9^−/−^(BALB/c.129)* mice. Once again, an age-associated increase in ANA titers was also detected in *Ly9^−/−^(BALB/c.129)* mice that was significantly higher than those of their age-matched wt counterparts. As was observed in the B6 background mice, the differences in ANA serum levels between wt and *Ly9^−/−^(BALB/c.129)* mice were statistically significant as early as 3 months of age, reaching its highest level at age 12 months when 91.6% of *Ly9^−/−^(BALB/c.129)* mice presented ANA titers ≥1:256 compared with 72.7% of their wt counterparts whose titers measured ≤1:64 (Figures 2A,B). Notably, ANA titers in *Ly9^−/−^(B6.129)* mice were always higher than those of *Ly9^−/−^(BALB/c.129)* at the evaluated time points, most likely due to the additional effect of the epistatic interactions, which induced spontaneous loss of immune tolerance to nuclear antigens (25). Comparable levels of anti-dsDNA and anti-nucleosome were detected on *Ly9*-deficient mice from both genetic backgrounds (Figures 2C,D and Figures 1C,D), although *Ly9^−/−^(B6.129)* mice displayed much higher ANA titers. This likely reflects the presence of preferential nuclear antigen recognition based on genetic background, which has been also observed in other lupus models (31).

**Figure 3:**
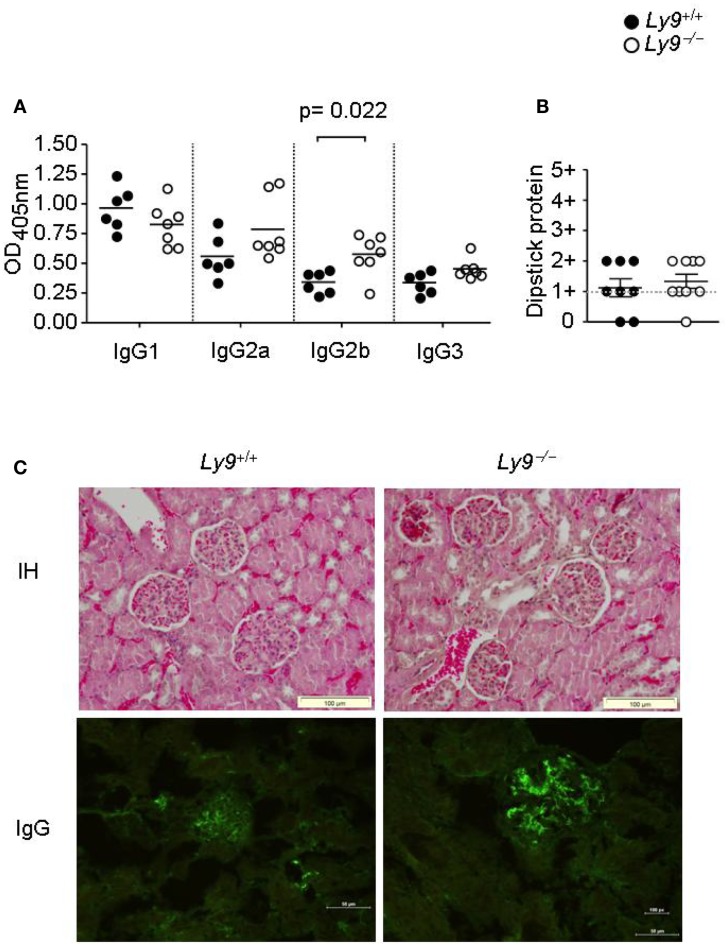
**One-year-old *Ly9^−/−^(BALB/c.129)* mice showed a skewed IgG isotype and no renal pathology**. **(A)** Indicated IgG isotypes were analyzed in sera from 12-month-old *Ly9^−/−^(BALB/c.129)* and wt mice. **(B)** Protein was measured with a colorimetric dipstick in fresh urine samples from 12-month-old mice. **(C)** Representative immunohistology (IH) image (Gill’s hematoxylin) of paraffin-embedded kidney sections (upper panels) and IgG-immunocomplex image detected by immunofluorescence (bottom panels) of cryopreserved kidney sections from 12-month-old wt (*n* = 4) and *Ly9*^−/−^ (*n* = 4) mice. Horizontal bars represent the mean level. SEM and statistical significance are shown.

*Ly9*-deficient mice exhibited a significant increase in IgG2b (Figure 3A) as well as higher ratios of IgG2a, 2b, and IgG3 isotypes vs. IgG1 than wt mice. Due to the high titers of autoantibodies observed on aged *Ly9*-deficient mice sera, we further investigated the presence of alterations in these animals’ renal physiology and functionality. Twelve-month-old *Ly9^−/−^(BALB/c.129)* mice did not exhibit proteinuria (Figure 3B) or differences in their glomeruli morphology compared to their wt counterparts. Although we observed a mild increase in IgG-immunocomplex deposits in *Ly9*-deficient mice (Figure 3C), this initial trait prior to any sign of glomerulonephritis did not trigger a renal pathology.

In summary, our findings reveal that the absence of the Ly9 receptor *per se* initiates the progressive development of autoantibodies, independently of any epistatic interactions.

### *Ly9^−/−^* aged mice exhibited splenomegaly and altered key cell subsets related to self-tolerance

**Figure 4:**
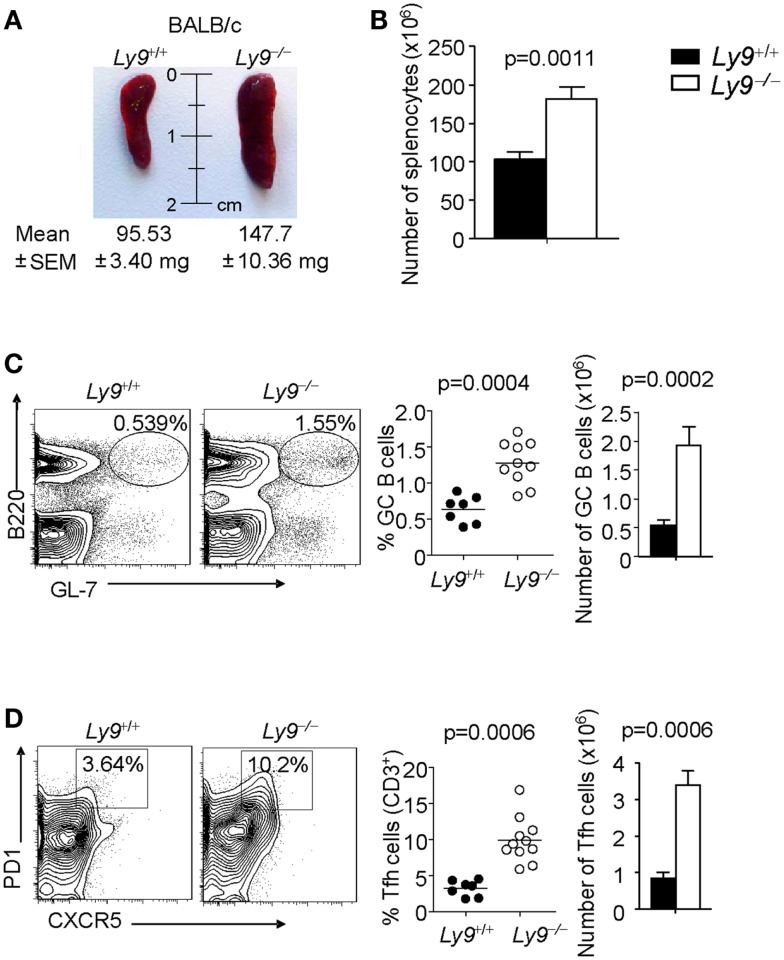
***Ly9* ablation alters key peripheral B and T subsets implicated in autoimmunity**. **(A)** Representative picture of spleens from 12-month-old wt (*n* = 7) and *Ly9^−/−^(BALB/c.129)* (*n* = 11); weight mean and SEM are shown. **(B)** Spleen cellularity was determined. **(C)** Splenocytes were stained with anti-B220 and anti-GL7 mAbs to calculate the percentages and total cell numbers in the germinal center (GC) B cell compartment, **(D)** or stained with anti-CD3, anti-CD4, anti-PD1, and anti-CXCR5 to analyze Tfh cells. Horizontal bars represent the mean level. SEM and statistical significances are shown.

The role of Ly9 as an inhibitor molecule in the development of spontaneous autoimmunity, excluding any effects dictated by epistatic interactions, is further supported by the observation that 12-month-old *Ly9^−/−^(BALB/c.129)* mice exhibited splenomegaly, which is a feature often present in SLE-prone mice (32, 33). The mean and SEM of spleen weight in wt (*n* = 7) and *Ly9^−/−^(BALB/c.129)* mice (*n* = 11) were 95.53 ± 3.40 and 147.7 ± 10.36 mg, respectively (*p* = 0.0021). Consistently, an increase of 75.8% in *Ly9^−/−^(BALB/c.129)* spleen cell numbers, compared with wt spleens, was observed (Figures 4A,B).

A deeper analysis of cell subsets by flow cytometry demonstrated altered B- and T-cell peripheral homeostasis in *Ly9*-deficient mice. We first inspected the B-cell signature of the disease in 12-month-old *Ly9^−/−^(BALB/c.129)* mice (Table 1). Notably, the most significant difference was found in GC B cells, with a percentage of 0.64 ± 0.19 in *wt* and 1.28 ± 0.30 in *Ly9*-deficient mice (*p* = 0.0004), which was also evident due the striking expansion of GC cell numbers (Figure 4C and Table 1). Although a major percentage of GC was observed in *Ly9*-deficient mice, only a slight decrease in the percentage of Follicular B cells was detected: 88.39 ± 3.15 (*Ly9*^+/+^) and 78.01 ± 7.25 (*Ly9^−/−^*), which is indicative of follicular B-cell areas with a major proportion of GC B cells. Transitional 1 (T1) B cells in wt and *Ly9^−/−^(BALB/c.129)* mice showed percentages of 4.48 ± 1.08 and 9.47 ± 3.12, respectively. Marginal zone (MZ) B cells from *Ly9*-deficient mice also showed increased percentages: 4.54 ± 1.78 (*Ly9*^+/+^) and 8.70 ± 2.97 (*Ly9^−/−^*) (Table 1). Thus, the previously mentioned B-cell subsets, most of them involved in autoimmune diseases (34, 35), showed a near twofold percentage increase in *Ly9^−/−^(BALB/c.129)* mice compared to their control counterparts.

**Table 1:** **Flow cytometry analysis of B- and T-splenic cell subsets from 12-month-old wt and *Ly9*^−/−^(BALB/c.129) mice**.

Parental	Subset	Marker	*Ly9*^+/+^		*Ly9*^−/−^
**B-CELL LINEAGE**					
B220^+^	T1	B220^+^CD21^−^CD23^−^	4.48 ± 1.08	**	9.47 ± 3.12
	Follicular-B	B220^+^CD21^+^CD23^high^	88.39 ± 3.15	**	78.01 ± 7.25
	MZ-B	B220^+^CD21^+^CD23^−/low^	4.54 ± 1.78	**	8.70 ± 2.97
Lymphocytes	GC B cells	B220^+^GL7^+^	0.64 ± 0.19	***	1.28 ± 0.30
Lymphocytes	Plasma B cells	B220^−/low^ CD138^+^	0.19 ± 0.03		0.24 ± 0.03
**T- AND NK-CELL LINEAGE**					
CD3^+^	CD4 SP	CD4^+^CD8^−^	73.55 ± 4.00		76.65 ± 5.56
	CD8 SP	CD4^+^CD8^+^	21.03 ± 4.08		16.28 ± 3.2
CD4^+^	Effector CD4	CD3^+^CD4^+^ CD8^−^	42.87 ± 4.26		50.50 ± 10.01
		CD44^high^CD62L^low^	
CD8^+^	Effector CD8	CD3^+^CD8^+^CD4^−^	17.53 ± 12.18		35.27 ± 15.02
		CD44^high^CD62L^low^	
CD4^+^	Reg T cells	CD3^+^CD4^++^CD25^+^	17.16 ± 1.62		20.10 ± 15.02
	Tfh	CD3^+^CD4^+^PD1^+^	3.27 ± 1.10	***	9.90 ± 3.09
		CXCR5^+^	
	Tefh	CD4^++^CD44^high^CD62L^low^	3.06 ± 1.42		5.29 ± 2.00
		CXCR5^−^PSGL-1^−^	
Lymphocytes	*i*NKT	B220^−^CD3^+^	3.53 ± 1.23	**	7.67 ± 2.8
		CD1d Tetr^+^	
CD3^−^	NK	CD3^−^DX5^+^	3.55 ± 1.02		3.60 ± 0.74

T1, transitional 1 B cells; MZ-B, marginal zone B cells; GC B cells, germinal center B cells; SP, single positive; Reg, regulatory; Tfh, follicular helper T cells; iNKT, invariant natural killer T cells; NK, natural killer; p-value significance; **p < 0.001, ***p < 0.0001. Results represent the mean ± SEM of 7 wt and 11 Ly9^−/−^(BALB/c.129) mice for each subset analyzed.

No altered proportion was found in the studied peritoneal B-cell subsets from the 12-month-old mice (data not shown). Our data also revealed a slightly increased frequency in the occurrence of the most immature B-cell lineages, multipotent progenitor (MMP) and Pro-B cells in the bone marrow of *Ly9*-deficient mice (data not shown), which is a variation that has been observed in other lupus-prone mice (36).

In examining the T-cell signature of *Ly9* deficiency-mediated autoimmunity, the most remarkable difference we observed was in Tfh cells, which showed a threefold percentage increase and cell number expansion (Figure 4D). Importantly, excessive Tfh-cell numbers have been linked to a positive-selection defect in GC, which would account for such differences in the autoantibody generation (21). As previously mentioned, *Ly9*-deficient mice also exhibited higher ratios of IgG2a,b and IgG3 isotypes vs. IgG1, suggesting that the peripheral tolerance checkpoint that controls GC and Tfh cells becomes altered by the absence of Ly9 molecule (Figure 3A). In the *Ly9*-deficient spleen, we found a slight increase in the ratios occurring between CD4^+^ and CD8^+^ T cells. We also observed an increase in effector CD4^+^ and CD8^+^ T-cell subsets, although it was not significant. Interestingly, the *i*NKT cell pools in these animals were also enlarged: 3.53 ± 1.23% wt and 7.67 ± 2.80% *Ly9^−/−^* mice (Table 1).

We conclude that *Ly9* gene ablation in a BALB/c background results in the disturbance of B and T cell subsets involved in autoimmunity, with major differences occurring in both Tfh cells and GC B cells.

### Ly9 receptor modulates IFN-γ secretion by CD4^+^ T cells

To determine the lymphocyte subsets that facilitate the ignition of SLE-related pathology in the absence of *Ly9*, we searched for any altered peripheral cell populations in 8- to 12-week-old *Ly9^−/−^(BALB/c.129)* mice. Slight differences were observed in the T-splenic compartment with a small increase in percentage of CD4^+^ T and *i*NKT. Notably, most of the alterations were displayed by B-cell subsets, with Transitional T1 B cells presenting the major difference (Table 2). A deeper examination of T1 and MZ B cells including IgM as a cell marker (T1:CD23^−^ CD21^−^ IgM^+^, MZ: CD23^−^ CD21^+^ IgM^+^) (37) demonstrates that *Ly9*-deficient mice possessed approximately a threefold increase of T1 subset and also an expanded MZ B subset (Figure S1 in Supplementary Material). Based on these results, we conclude that *Ly9*-deficiency alters the development of B-cell subsets which may be involved in the generation of autoantibodies.

**Table 2:** **Flow cytometry analysis of B- and T-splenic cell subsets from 8- to 12-week-old wt and *Ly9*^−/−^(BALB/c.129) mice**.

Parental	Subset	Marker	*Ly9*^+/+^		*Ly9*^−/−^
**B-CELL LINEAGE**					
B220^+^	T1	B220^+^CD21^−^CD23^−/low^	10.87 ± 0.71	***	15.18 ± 0.46
	Follicular-B	B220^+^CD21^+^CD23^high^	79.96 ± 0.84	***	74.30 ± 0.18
	MZ	B220^+^CD21^+^CD23^−/low^	4.76 ± 0.21	*	5.79 ± 0.31
Lymphocytes	GC B cells	B220^+^GL7^+^	4.65 ± 0.65		4.25 ± 1.04
Lymphocytes	Plasma B cells	B220^−/low^ CD138^+^	0.27 ± 0.06		0.22 ± 0.08
**T- AND NK-CELL LINEAGE**					
CD3^+^	CD4 SP	CD4^+^CD8^−^	65.93 ± 6.27	*	71.53 ± 1.57
	CD8 SP	CD4^−^CD8^+^	16.81 ± 4.78		12.7 ± 1.53
CD4^+^	Effector CD4	CD3^+^CD4^+^CD8^−^	28.58 ± 1.66		29.80 ± 1.13
		CD44^high^CD62L^low^	
CD8^+^	Effector CD8	CD3^+^CD8^+^CD4^−^	7.50 ± 2.80		8.26 ± 1.55
		CD44^high^CD62L^low^	
CD4^+^	Reg T cells	CD3^+^CD4^+^CD25^+^	8.77 ± 1.22		9.33 ± 0.76
	Tfh	CD3^+^CD4^+^PD1^+^	3.40 ± 0.44		3.55 ± 0.54
		CXCR5^+^	
Lymphocytes	*i*NKT	B220^−^CD3^+^	4.22 ± 0.53	*	5.11 ± 0.69
		CD1d Tetr^+^	
CD3^−^	NK	CD3^−^DX5^+^	0.82 ± 0.32		0.63 ± 0.14

T1, transitional 1 B cells; MZ-B, marginal zone B cells; GC B, germinal center B cells; SP, single positive; Reg, regulatory; Tfh, follicular helper T cells; iNKT, invariant natural killer T cells; NK, natural killer; p-value significance; *p < 0.05, ***p < 0.0001. Results represent the mean ± SEM of wt and Ly9^−/−^(BALB/c.129) mice (n = 5) for each subset analyzed.

**Figure 5:**
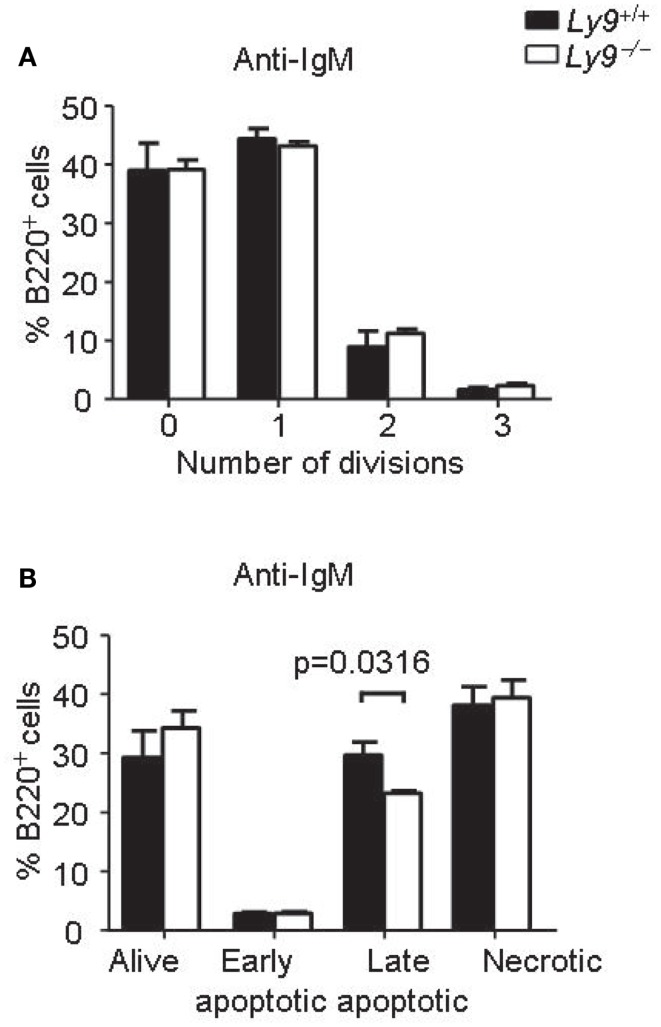
***Ly9*-deficient B cells displayed similar proliferation and survival than wt cells after IgM stimulation**. Splenic cells were obtained from *Ly9^−/−^(BALB/c.129)* (*n* = 4) or wt (*n* = 4) mice and stimulated with anti-IgM antibody during 72 h. **(A)** The cells were labeled with CFSE. Flow cytometry analysis of gated B220^+^ cells was performed. The percentages of B cells in each division are shown. **(B)** Apoptosis assay on gated B220^+^ cells was assessed by flow cytometry. The cells were stained with Live/dead fixable far red and Annexin V eFluor 450. Quantification of the data is presented in a bar diagram (Alive; Live/dead^−^ Annexin V^−^, early apoptotic; Live/dead^−^ Annexin V^+^, late apoptotic; Live/dead^+^ Annexin V^+^, necrotic; Live/dead^+^ Annexin V^−^). **(A,B)** are representatives of two independent experiments.

Abnormalities in BCR signaling could shape the splenic B-cell populations and predispose to autoimmune disease. Therefore, we assessed the proliferation and survival of splenic B cell from *Ly9*^−/−^ mice after IgM stimulation. The proliferation and apoptotic responses of *Ly9*-deficient B cells were similar to those observed in the wt B cells, with the exception of a slight decrease in percentage of late apoptotic cells in the *Ly9*-deficient mice (Figure 5).

**Figure 6:**
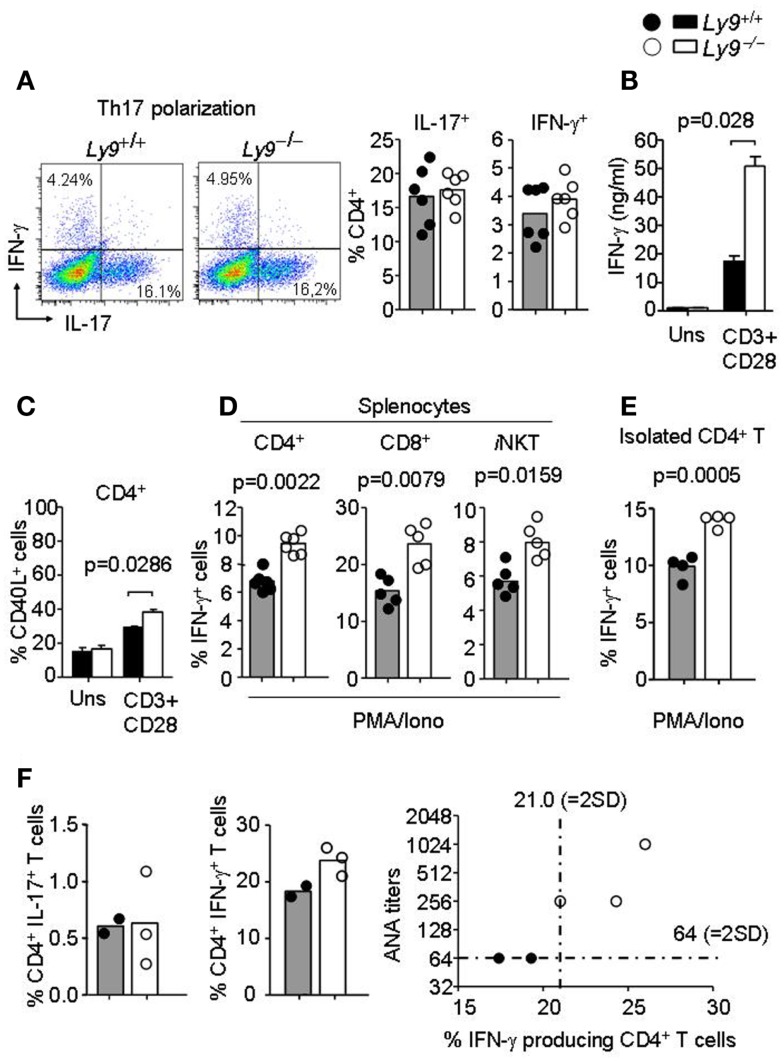
***Ly9*-deficient mice foster skewed IFN-γ producing T and *i*NKT cell populations prior to full disease onset**. **(A)** Representative dot plot histogram of IFN-γ and IL-17 producing CD4^+^ T cells from 3-months-old *Ly9*^−/−^*(BALB/c.129)* or wt mice splenocytes after exposure to Th17 polarizing conditions. Bar histograms show percentage of IL-17 and IFN-γ producing CD4^+^ T cells, respectively. **(B)** Amounts of IFN-γ in the culture supernatants of 72 h anti-CD3 (2 μg/ml) and anti-CD28 (1 μg/ml) mAb-stimulated splenocytes as measured by ELISA (*n* = 4 mice per group). **(C)** Histogram of CD40L expression in CD4^+^ T cells after 24 h activation, as judged by flow cytometry analysis (*n* = 4 mice per group). **(D)** Percentage of IFN-γ producing CD4^+^ and CD8^+^ T, and *i*NKT gated from total splenocytes or **(E)** isolated splenic CD4^+^ T cells from 8- to 12-week-old *Ly9^−/−^(BALB/c.129)* or wt mice as analyzed by flow cytometry after 4 h cell activation with PMA, ionomycin, and GolgiStop. **(F)** Splenocytes from 1-year-old mice were activated with PMA, ionomycin, and GolgiStop and then IL-17 and IFN-γ were analyzed by flow cytometry. Bar histograms show the percentage of CD4^+^ T cells producing IL-17 and IFN-γ. Correlation between ANA titers and percentage of IFN-γ producing CD4^+^ T cells is represented in a distribution histogram; dotted line represents mean of ANA titers + (2×) standard deviation (SD) or mean percentage of IFN-γ producing CD4^+^ T cells + (2×) SD from wt mice. Horizontal bars represent the mean level. Statistical significances are shown. Data from **(A,D,E)** are representative of two independent experiments. Data from **(B,C)** are representative of three independent experiments.

Since earlier studies have demonstrate the critical implication of SLAMF receptors in Th1/Th2/Th17 polarization (14, 26, 38), we hypothesized that *Ly9*-deficient splenocytes in 8- to 12-week-old mice would foster an alteration in T-cell cytokine production prior to disease onset, thereby enabling autoantibody production at older ages. First, we asked whether the ablation of murine *Ly9* could modulate IL-17 secretion by CD4^+^ T cells under Th17 polarizing conditions, since earlier reports have implicated the human Ly9 receptor in IL-17 T-cell secretion (38, 39). No significant differences were detected in the percentage of IL-17 secreting CD4^+^ T cells (Figure 6A). On the other hand, our group has previously shown that monoclonal antibodies against Ly9 negatively regulate TCR signaling, thereby inhibiting ERK phosphorylation and IFN-γ secretion (26, 40). Herein, we investigated the role of Ly9 in IFN-γ modulation by activating splenic T cells with anti-CD3 and anti-CD28. A significant increase in IFN-γ secretion, as well as an increase in the expression of CD40L in CD4^+^ T cells was detected in the absence of Ly9 compared with wt cells (Figures 6B,C). A similar result was obtained when evaluating the expression of the CD25 activation marker (data not shown). In addition, PMA/ionomycin activation of splenic cells revealed that *Ly9*-deficient mice foster CD4^+^ T, CD8^+^ T, and *i*NKT cells capable of secreting major quantities of IFN-γ prior to the development of autoimmunity (Figure 6D). Furthermore, isolated *Ly9-*deficient CD4^+^ T cells consistently showed an increased IFN-γ production after PMA/ionomycin activation as compared with wt mice (Figure 6E). In contrast, no significant difference in percentage of IL-4 producing CD4^+^ T cells was observed (data not shown). In accordance with these results, 12-month-old *Ly9*^−/−^*(BALB/c.129)* mice exhibited an increased percentage of IFN-γ producing CD4^+^ T cells, which correlated with the high ANA titers detected in serum (Figure 6F). Nevertheless, we could not detect IFN-γ in the serum of these mice (data not shown).

Thus, in the absence of the Ly9 molecule occurs an increase in peripheral T and *i*NKT IFN-γ secretion, a key cytokine in the pathogenesis of SLE which has been previously shown to trigger the accumulation of pathogenic Tfh and GC B cells (41).

## Discussion

Despite extensive research, the mechanisms by which susceptibility and effector genes initiate and promote autoantibody production and tissue damage remain poorly defined. In the present study, we have shown that *Ly9* deficiency results in a spontaneous loss of tolerance, reflected principally in the development of autoantibodies, a process which is thought to underlie the initiation of SLE syndrome.

We first found that the absence of the *Ly9* gene in mice with a B6 and BALB/c background resulted in the development of autoantibodies. The loss of tolerance to nuclear antigens detected in *Ly9^−/−^(B6.129)* mice was reminiscent of those previously observed in B6.Sle1b and B6.129Chr1b congenic mouse strains (6, 42). We found that *Ly9^−/−^(B6.129)* mice developed autoantibodies as early as 12 weeks of age. In fact, by 9 months of age all mice were autoantibody positive, a pattern similar to that found in SLAMF receptor *Slamf1*-deficient *(B6.129)* mice (25). Most *SLAMF receptor*-deficient mice have been generated by altering those *SLAMF genes* located in chromosome 1 via the homologous recombination of *129*-derived embryogenic stem cells (25, 29, 43, 44). Once the resultant mice are backcrossed onto B6 mice [e.g., *Slamf1*^−/−^*(B6.129)*], the deficient mice are affected by the epistatic interaction that occurs between the 129 gene segment and the B6 genome. Thus, epistatic interactions between 129-derived and B6 genes in *Ly9^−/−^(B6.129)* mice greatly contribute to the autoantibody response. Exceptionally, the disruption of the *Ly108* gene in congenic mice *[Ly108^−/−^(B6.129)]* mitigates the generation of autoantibodies, indicating that SLAMF members carry out opposing functions (e.g., Ly108 vs. Ly9).

We next assessed the role of Ly9 in autoimmune disease without the confounding influence of mixed haplotypes by utilizing *Ly9^−/−^(BALB/c.129)* mice. Although autoantibody titers were lower compared to *Ly9^−/−^(B6.129)* mice, this strain clearly developed an autoimmune response based on the significant increases of ANA, anti-dsDNA, and anti-chromatin antibodies compared to their wt counterparts. By contrast, other *SLAMF receptor*-deficiencies embedded in the BALB/c genome, such as *Slamf1^−/−^(BALB/c.129)* and *Slamf2^−/−^(BALB/c.129)*, do not develop any autoimmune response (25), underscoring the role played by Ly9 as a negative regulator in the pathogenesis of lupus. Consequently, among the various SLAMF receptors, both Ly9 and 2B4 rise as unique factors contributing to the reinforcement of tolerance (24).

As a consequence of breaching tolerance, a plethora of disorders can develop *a posteriori*; e.g., T and B cells are reportedly involved in the amplification and perpetuation of the autoimmune response, resulting in inflammation and cytokine dysregulation (45). This proved to be the case in our 12-month-old *Ly9*-deficient mice in which various B and T cells subsets underwent alterations. Notably, the most noteworthy differences in older mice were found in Tfh and GC B cells, two cell types which have been shown to preferentially express the Ly9 receptor (46).

In order to begin to understand why *Ly9*-deficient mice developed spontaneous autoimmunity, we search for any abnormal peripheral B and T cell development prior to full autoantibody disorder on 8- to 12-week-old *Ly9*-deficient mice. We observed alterations in the B-cell splenic compartment, with the most prominent expansion displayed by *Ly9*-deficient transitional T1 B cells. Interestingly, SLE patients present increased numbers of T1 cells, although their role in lupus is still ill defined (35). In addition, 8- to 12-week-old *Ly9*-deficient mice displayed an increase in MZ B cells. Even though many autoreactive antibodies appear to be the product of GC reactions, major evidence begins to reveal that MZ B cells play a key role in homeostasis and tolerance. Notably, the MZ B-cell expansion has been directly implicated in lupus pathogenesis in some murine models (47–50), but not others (51, 52). Autoimmunity mediated by B cell is usually linked to a B-cell hyperresponse which change the B-cell splenic composition. Thus, an increased BCR signal, as shown by mice deficiencies in SHP-1, FcγRII, CD22, Cbl-b, or overexpressing CD19, leads to a B-cell hyperresponsiveness upon BCR stimulation, change B-cell subsets normal architecture, and culminates in a systemic autoimmune disease (53). Here, we show that there’s similar proliferation and survival of *Ly9*-deficient B cells as compared with wt cells, although we can not exclude the abnormal function of particular B-cell subsets in the *Ly9*-deficient mice which could be the subject of future investigations.

Next, we investigated how the absence of Ly9 could possibly contribute to a cytokine imbalance prior to disease onset. Others have shown that the engagement of human naive CD4^+^ T lymphocytes with an anti-human Ly9 monoclonal antibody under Th17 polarizing conditions results in an increase in IL-17 (38, 39). No alterations in IL-17 producing T lymphocytes were observed once *Ly9-*deficient mice splenic cells were activated under Th17 polarizing conditions. Previous reports showed a diminishing IL-4 production and no altered IFN-γ secretion by *Ly9*-deficient CD4 T cells (29). In contrast, in the absence of Ly9, we observed an increase in IFN-γ producing T cells with no significant alteration in IL-4 secretion. Importantly, IFN-γ has long been associated with lupus (54). This apparent contradiction could be explained by the influence of epistatic interactions as well as the mice background, since previous studies were performed in *Ly9^−/−^* with a mixed background (B6 × 129) mice. Recent reports have demonstrated that the overproduction of IFN-γ induces an aberrant accumulation of Tfh and GC cells (41).We also found that these subsets underwent expansion in

*Ly9*-deficient mice, when the influence of confounding epistatic interactions was absent. These observations suggest that the Ly9 molecule may play an inhibitory role in the expansion of these subsets. In addition, *Ly9*-deficient mice showed a skewed isotype switching toward IgG2a/b, an isotype induced by a Th1 response that requires T cell-stimulated B lymphocytes. Although IgG2a antibodies are the most pathogenic class of immunoglobulin (55, 56), we did not observe any major kidney pathology. However, a mild increase in IgG-immunocomplex deposits was detected in *Ly9*-deficient mice, a stage that often precedes full-blown kidney pathology. Thus, an *Ly9* mutation confers a predisposition to autoantibody generation, but one that may require additional factors (e.g., a viral infection or additional gene alterations) before a full lupus disease pathogenesis becomes evident. In addition, we cannot exclude the influence of *Ly9*-deficient *i*NKT cells, a population that underwent expansion in 8- to 12-week-old *Ly9^−/−^* mice (30), on the observed autoimmune response. Although *i*NKT cells generally play a tolerogenic role in autoimmunity, some models, and depending on the mouse strain and treatment protocol used, *i*NKT cell activation exacerbates rather than protects against autoimmunity (57), e.g., the stimulation of *i*NKT cells triggers a Th1 immune response in adult NZB/W mice, resulting in the exacerbation of systemic autoimmunity (58). Thus, *i*NKT disease protection is associated with enhanced Th2 and/or reduced Th1 responses against targeted antigens. A deeper study of *Ly9*-deficient *i*NKT cell will be needed to determine the specific role of this cell-type in the observed autoimmune phenotype.

In summary, the data presented in this study sheds light on the inhibitory function of the Ly9 cell-surface receptor, suggesting that this molecule is involved in the maintenance of peripheral cell tolerance by serving as a negative regulator of the immune response. Further investigation will be required to elucidate the precise mechanism by which Ly9 confers protection from autoimmunity. Nonetheless, this study demonstrates that Ly9 may not only prove to be a valuable target for the treatment of autoimmunity, but may also help build a more rational road map toward understanding the molecular causes of SLE syndrome.

## Conflict of Interest Statement

The authors declare that the research was conducted in the absence of any commercial or financial relationships that could be construed as a potential conflict of interest.

## Supplementary Material

The Supplementary Material for this article can be found online at http://www.frontiersin.org/Inflammation/10.3389/fimmu.2013.00225/abstract

Supplementary Figure S1 **Splenic T1 and MZ cells are expanded in *Ly9^−/−^(BALB/c.129)* mice**. **(A)** Spleen lymphocytes from 8- to 12-week-old wt (*n* = 5) and *Ly9^−/−^(BALB/c.129)* mice (*n* = 5) were stained using CD23, CD21, and IgM. **(A)** Representative dot plots from wt and *Ly9^−/−^* splenic cells. The gating strategy to characterize transitional and marginal zone (MZ) B cells is shown. **(B)** Quantitative analysis of the CD23^−^ percentage of MZ and Transitional 1 (T1) B cells as well as **(C)** MZ- and T1-B cells cellularity per spleen are shown. SEM and statistical significances are shown.
