# Supplementary figures and images for: Ly9 (CD229) Cell-Surface Receptor is Crucial for the Development of Spontaneous Autoantibody Production to Nuclear Antigens
Source: Front Immunol. 2013 Jul 31;4:225. doi: 10.3389/fimmu.2013.00225 (PMC3728625; doi:10.3389/fimmu.2013.00225)

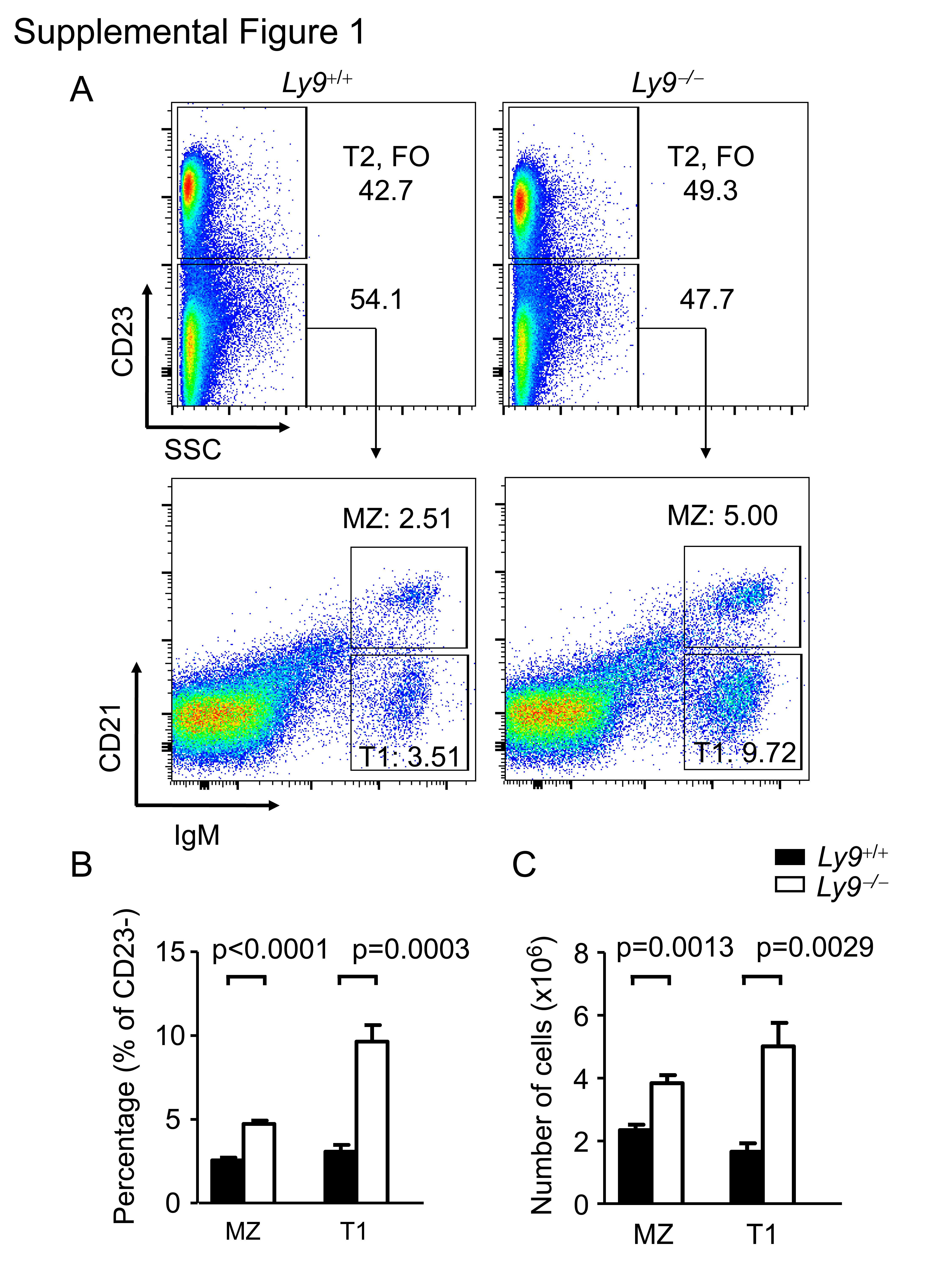

Supplement: Supplementary Figure S1 — Splenic T1 and MZ cells are expanded in Ly9−/−(BALB/c.129) mice. (A) Spleen lymphocytes from 8- to 12-week-old wt (n = 5) and Ly9−/−(BALB/c.129) mice (n = 5) were stained using CD23, CD21, and IgM. (A) Representative dot plots from wt and Ly9−/− splenic cells. The gating strategy to characterize transitional and marginal zone (MZ) B cells is shown. (B) Quantitative analysis of the CD23− percentage of MZ and Transitional 1 (T1) B cells as well as (C) MZ- and T1-B cells cellularity per spleen are shown. SEM and statistical significances are shown. [file 56070_Romero_Presentation1.ZIP › Supplementary figure/Supplemental Fig 1.tif]
